# Bio-Inspired 3D-Printed Polymeric Sheets for Orthoses: Predictive Modeling of Mechanical Integrity and Moisture Absorption

**DOI:** 10.3390/biomimetics11060417

**Published:** 2026-06-13

**Authors:** Rosa Devesa-Rey, Elena Arce, Silvia Losada-Pérez, Miguel Ángel Álvarez-Feijoo, Raquel Leirós-Rodríguez

**Affiliations:** 1 University Defense Center at Spanish Naval Academy, University of Vigo, 36920 Marín, Spain; 2 Department of Industrial Engineering, Ferrol Polytechnic School of Engineering, University of A Coruña, 15403 Ferrol, Spain; 3 Department of Design, School of Industrial Engineering, University of Vigo, 36310 Pontevedra, Spain; silviamaria.losada@uvigo.gal (S.L.-P.); alvarezfeijoo@uvigo.gal (M.Á.Á.-F.); 4 SALBIS Research Group, Nursing and Physical Therapy Department, University of León, 24401 Ponferrada, Spain; rleir@unileon.es

**Keywords:** additive manufacturing, orthotic devices, saline degradation, Box–Behnken design, polylactic acid, Shore hardness

## Abstract

The rapid development of additive manufacturing has enabled the production of personalized biomedical devices, including custom orthoses that must retain their structural integrity under demanding physiological conditions. This study evaluates the performance of 3D-printed polymers—blue and white polylactic acid (PLA), Standard Blue Resin, and an ecological soy-based resin—after exposure to simplified, controlled saline environments related to sweat contact and hygiene-associated conditions. Moisture absorption and Shore A hardness were analyzed as response variables to assess material stability under different experimental conditions. A surface methodology based on a Box–Behnken design was used to quantify the effects of specimen thickness (x_1_), NaCl concentration (x_2_), and immersion time (x_3_) on the selected dependent variables. The results indicate that Standard Blue Resin showed the greatest surface hardness stability, whereas the bio-based materials (PLA and ecological resin) were more susceptible to moisture absorption, particularly in thinner polymeric sheets. The fitted quadratic models provide a predictive framework for optimizing material selection and geometric design in biomimetic wearable devices, supporting the development of orthoses with improved durability, hygiene, and long-term functional performance.

## 1. Introduction

Between 35 and 40 million people—approximately 0.5% of the world’s population—require prosthetics, orthotics, and rehabilitation treatments. This demand is expected to double by 2050 [1]. According to the World Health Organization (WHO), it is estimated that only about 5% to 15% of people who could benefit from assistive devices have access to appropriate devices, such as prostheses and orthoses [2].

Foot orthotics are custom-made devices designed for each individual that are placed inside shoes to support, align, prevent, and correct orthopedic deformities, or improve foot and ankle function by distributing weight [3,4]. The main objective of these devices is to reduce pain, correct postural defects, prevent injuries, or manage various types of gait pathologies through the efficient distribution of weight across the sole of the foot [5,6,7].

The fabrication of this type of orthosis requires biomechanical analysis (static and dynamic) [8] and significant hands-on manufacturing time [9], using precise molds (plaster, foam, or digital) to adapt the device to the patient’s specific condition and type of footwear, thereby ensuring an optimal fit and effective therapeutic results [1,8,10]. These types of devices have existed for many centuries and are made using traditional materials and techniques [11]. In traditional manufacturing, a patient who needs a prosthesis or orthosis must visit a prosthetist or orthotist to have anthropometric measurements taken. The affected part of the body is wrapped in plaster bandages to create a negative cast, which is then used to make a positive cast by pouring plaster into the negative cast. The next step is to manufacture the prosthesis or orthosis by heating and vacuum-forming sheets of thermoplastic (usually polypropylene or polyethylene) over the positive plaster mold; these are allowed to cool and then trimmed to the correct shape. It may be necessary to modify the plaster mold or add an additional component, depending on the load borne by the sensitive and weight-bearing areas of the human body. Next, the necessary accessories and/or straps are added to ensure that the device is properly secured. In most cases, additional adjustments are required to ensure the comfort and functionality of the product. These traditional processes generate a significant amount of material waste and involve considerable time and labor, with associated costs [12]. Very importantly, the quality of the products depends largely on the skill and experience of the prosthetist or orthotist [13,14].

In the 1970s, new techniques were developed in response to the demand for orthopedic devices with a more attractive appearance, such as plastic coatings achieved by applying a tinted rubber-based plastic film [15]. This development improved the visual appearance and comfort of orthoses. In the early 1980s, the rise in additive manufacturing (AM) technologies—popularly known as 3D printing technologies in the manufacturing sector—with the introduction of stereolithography, a technique based on curing photopolymer resin in thin layers with a UV laser, enabled the construction of 3D models [16]. In the following years, other technologies were introduced. Fused Deposition Modeling (FDM) [17,18] and Vat Photopolymerization (SLA/DLP) are the most prominent, utilizing thermoplastic filaments and photopolymer resins, respectively, to create high-resolution functional prototypes. Other technologies include Laminated Object Manufacturing (LOM), Selective Laser Sintering (SLS), 3D printing, and variable rapid prototyping (Polyjet Technology) [19]. Additive Manufacturing (AM) has revolutionized the production of customized biomedical devices, particularly in the field of orthotics and prosthetics [20,21]. Unlike traditional subtractive manufacturing, AM allows for the layer-by-layer fabrication of complex geometries that can be tailored to the specific anatomical needs of a patient. AM technologies are included in the field of rapid prototyping techniques, which can produce fully functional parts directly from a three-dimensional model without a machining process [22,23]. This capability is fundamental in biomimetic design, where the goal is to replicate the functional efficiency and structural hierarchy found in natural tissues [24,25]. These advances in the field of biomedical engineering have also occurred at a rapid pace due to the need to create specific, customized devices capable of adapting properly to the patient’s anatomy [26,27,28,29]. For this reason, these rapid prototyping techniques are particularly valuable in the orthopedic and prosthetic industry, where devices must fit the patient’s body perfectly to fulfil their rehabilitative function and prevent rejection by the patient, as many of them cause blisters, ulcers, or discomfort [30]. Rapid prototyping is already used in the manufacture of spinal braces [31], exoskeleton components [32], and passive orthoses [33,34], as well as in the medical and dental industries [19]. They have spread rapidly due to the high quality of the products, short lead times, and competitive costs [35].

The first step in the manufacturing process begins with capturing the subject’s morphology using 3D scanning technologies. Next, computer-aided design (CAD) and computer-aided engineering (CAE) tools are used to create designs tailored to each individual. Meanwhile, functionality is evaluated by testing different materials and structures. Finally, the design is exported to an additive manufacturing machine to produce the prototype. Manufacturing time can range from several weeks in the traditional process to a couple of days with rapid prototyping [16]. In the field of 3D printing, FDM is a technique with great potential because it allows for the use of low-cost materials. In this process, a semi-molten material is extruded through a moving extrusion head, creating two-dimensional layers of the part to be manufactured. Once a layer is complete, the head moves up, and another layer is extruded. The process continues layer by layer until the part is finished. This extrusion head has an extrusion nozzle to deposit the material for the part, but it may also have another nozzle to deposit a different material that acts as a support [36].

The most common materials used in this process are polycarbonate (PC) and acrylonitrile butadiene styrene (ABS), or a mixture of both. These materials have properties similar to those of thermoplastics used in injection molding. However, other materials, such as thermoplastics, polymers or nylon-based materials, are also used today [37,38].

The selection of materials for orthotic applications is critical. Thermoplastics like Polylactic Acid (PLA) are favored for their ease of use and biodegradability [39], while resins are valued for their superior isotropic properties, high surface finish, and mechanical precision [40]. These materials must not only provide structural support but also withstand the demanding conditions of the human physiological environment. Orthoses are in constant contact with the skin, exposing them to human sweat—a complex solution of water, minerals (primarily sodium chloride), and metabolic byproducts. Furthermore, these devices often require frequent cleaning and disinfection with saline or chemical solutions to maintain hygiene and prevent microbial proliferation.

Despite their advantages, 3D-printed polymers are susceptible to environmental degradation. The hygroscopic nature of many printing filaments and the chemical structure of certain resins make them prone to moisture absorption [41]. This phenomenon can lead to plasticization, swelling, and a subsequent decline in mechanical integrity, such as changes in Shore hardness and tensile strength. In the context of orthotics, any significant alteration in material properties could compromise the device’s corrective function or cause discomfort to the user. Therefore, characterizing the long-term resilience of these materials in saline and humid environments is essential for ensuring their clinical viability [42].

To address this challenge, systematic optimization and predictive modeling are required. Design of Experiments (DoE) methodologies, such as the Box–Behnken method, provide robust frameworks for analyzing the influence of multiple variables—such as material thickness, salt concentration, and exposure time—on the performance of 3D-printed parts [43]. The Box–Behnken design allows for a more detailed exploration of non-linear interactions through response surface methodology [44]. Recent advances in biomimetic and additively manufactured biomaterials further support the need to combine material selection, structural design, and durability assessment in biomedical devices. For example, additive manufacturing has been increasingly explored for biodegradable metallic biomaterials, where material composition, manufacturing quality, degradation behavior, and biocompatibility are key research challenges. In parallel, bio-inspired strategies based on microporous architectures and natural interfaces, such as insect cuticles, have shown how structural design can improve toughness, elasticity, adaptability, and durability. These recent studies highlight the importance of integrating biomimetic design principles with predictive material evaluation when developing 3D-printed orthotic components [45,46,47].

This study applies a Box–Behnken design within the response surface methodology framework to evaluate the influence of specimen thickness, NaCl concentration, and immersion time on moisture absorption and Shore A hardness of 3D-printed polymers for biomedical orthoses.

This study aims to evaluate and compare the predictive capabilities of the Box–Behnken model in assessing the behavior of 3D-printed polymeric sheets under saline conditions. By analyzing moisture absorption and hardness stability in various resins and PLA filaments, this research provides a scientific basis for the selection and design of durable, bio-inspired orthotic devices capable of maintaining their functionality under controlled saline exposure conditions relevant to orthotic use.

## 2. Materials and Methods

### 2.1. Experimental Materials and Methods

#### 2.1.1. Material Selection and Biomimetic Relevance

The selection of materials focused on two distinct categories of polymers widely used in the medical and industrial sectors: photopolymerizable resins and thermoplastic filaments. Four commercial polymers were selected to evaluate their performance in personalized orthotics. The selection criteria focused on the balance between mechanical versatility and environmental sustainability.

On the one hand, two different types of thermoplastic filaments (FDM) were chosen, namely blue and white polylactic acid (PLAB and PLAW). PLA is a bio-based, biodegradable polymer derived from renewable resources (corn starch or sugar cane), making it a primary candidate for sustainable biomimetic design. On the other hand, two types of photopolymer resins (SLA/DLP) were also employed, namely a standard blue resin (Std-Resin) and ABS-like Resin Plus, an ecological resin (Eco-Resin) (Elegoo, Shenzhen, China). Std-Resin is a general-purpose resin suitable for a wide variety of applications, whereas ABS-like Eco-Resin Plus mimics the mechanical properties of ABS and is ideal for 3D printing parts that require high strength and durability. In addition, the inclusion of an ecological—soy-based—resin is crucial for assessing how green alternatives respond to chemical degradation compared to traditional synthetic resins.

#### 2.1.2. Specimen Manufacturing

Two additive manufacturing techniques were used according to the material type. PLA specimens were manufactured by Fused Deposition Modeling (FDM) (Creality Ender; Shenzhen Creality 3D Technology Co., Ltd., Shenzhen, China), and resin specimens were manufactured by VAT photopolymerization (Elegoo Mars 3; Elegoo, Shenzhen, China). This methodology is particularly advantageous for the development of personalized orthotic devices, where anatomical precision is crucial.

The specimens were designed as polymeric sheets (12.7 × 12.7 cm) using AutoCAD Inventor, with thicknesses varying between 2 mm and 6 mm to simulate different structural requirements of an orthosis. Thickness was selected as the main geometrical variable as it affects moisture diffusion and mechanical response in thin orthotic specimens. Other variables, such as specimen length, surface area or geometry, were kept constant to isolate the effect of thickness. To ensure the scientific validity of the moisture absorption tests, specific printing parameters were controlled: (i) All FDM samples were printed with 100% solid infill. This is a critical methodological step to ensure that any weight gain recorded is due to the intrinsic hygroscopy of the polymer and not to the mechanical entrapment of fluid within internal lattice voids or air pockets. (ii) Resin specimens were cleaned in isopropyl alcohol and subjected to a controlled UV post-curing cycle for 20 min. This ensures the maximum degree of monomer conversion, stabilizing the chemical structure before exposure to saline environments.

#### 2.1.3. Experimental Setup

Three independent variables were considered: saline concentration (NaCl, 0.1–5%), exposure time (1–6 days), and specimen thickness (2–6 mm). Their effects were evaluated on the hardness and moisture content of the two resins (Eco-Resin and Std-Resin) and the two PLA specimens (PLA_B_ and PLA_W_). Distilled water (0% NaCl) was used as the control condition. The selected conditions were intended to provide a simplified approximation of sweat-related saline exposure and hygiene-associated contact, rather than a complete reproduction of real orthotic operating conditions, both of which are relevant to the clinical durability of wearable medical devices. Therefore, the NaCl concentration range was selected to cover both physiologically relevant and accelerated saline-stress conditions. Human sweat composition is highly variable, with reported sweat sodium concentrations typically ranging from approximately 10 to 90 mmol/L, depending on individual, environmental, and activity-related factors. Standardized and commonly used artificial sweat formulations also include NaCl as a major ionic component, with concentrations varying from 0.5% to 1% NaCl [48,49,50] in artificial perspiration solutions. NaCl solutions were used as simplified saline media to evaluate the isolated effect of salt concentration. However, it must be remarked that they do not fully reproduce physiological sweat composition; therefore, the results should be interpreted as controlled saline exposure tests. The lower and intermediate levels used in this study were selected to represent sweat-related exposure, whereas the 5% NaCl level was included as an accelerated saline challenge, consistent with the concentration commonly used in neutral salt-spray corrosion testing. The exposure time range of 1–6 days was selected to evaluate both short-term and cumulative exposure [51].

Surface hardness was measured using a calibrated Shore A durometer [52]. This test measures the resistance of a material to indentation. Shore A hardness provided measurable and reproducible values within the tested material range, enabling the assessment of relative surface hardness changes after saline exposure. Three measurements were taken per specimen to reduce the influence of local variability and obtain a representative mean value of material rigidity. The arithmetic mean and standard deviation were calculated for the final Shore A hardness value.

Moisture content was also determined in triplicate following the guidelines of ISO 15512 [53]. Before weighing, specimens were placed in an oven at 45 °C for 40 min to remove superficial moisture without altering the internal properties of the polymer.

#### 2.1.4. Experimental Design and Statistical Optimization

A Design of Experiments (DoE) approach based on the Box–Behnken design (BBD) was used to generate response surfaces using a three-level fractional factorial design. BBD comprises a set of mathematical and statistical techniques based on fitting empirical models to experimental data obtained from experimental designs [54]. Box–Behnken designs are rotatable or nearly rotatable second-order designs based on incomplete three-level factorial designs [55]. The simplest equation describing a linear function is described by Equation (1).

(1) $$y=\beta_{0}\sum_{i=1}^{k}\beta_{i}X_{i}+\varepsilon$$

where *β_0_* is the constant term, *β_i_* represents the coefficients of the linear parameters, *k* is the number of variables, *x_i_* represents the independent variables, and *ε* is the residual error associated with the experiments [56]. When experimental data do not fit a linear equation, because the optimum response is not necessarily located at the highest or lowest values of the variables, higher-order terms must be included in the model. In such cases, a polynomial response surface is generated. Box–Behnken designs are constructed to fit a second-order model, as shown in Equation (2).

(2) $$y=\beta_{0}\sum_{i=1}^{k}\beta_{i}X_{i}+\sum_{i=1}^{k}\sum_{j\geq i}^{k}\beta_{ij}X_{i}X_{j}+\varepsilon$$

where *βij* represents the coefficients of the interaction parameters. These designs include a central point that is used to assess curvature. Critical or optimum conditions are obtained from the second-order function by including quadratic terms, as shown in Equation (3).

(3) $$y=\beta_{0}\sum_{i=1}^{k}\beta_{i}X_{i}+\sum_{i=1}^{k}\beta_{ii}X_{i}^{2}+\sum_{i=1}^{k}\sum_{j\geq i}^{k}\beta_{ij}X_{i}X_{j}+\varepsilon$$

(4) $$y=\beta_{0}+\beta_{1}x_{1}+\beta_{2}x_{2}+\beta_{3}x_{3}+\beta_{12}x_{1}x_{2}+\beta_{23}x_{2}x_{3}+\beta_{23}x_{2}x_{3}+\beta_{11}x_{1}^{2}+\beta_{22}x_{2}^{2}+\beta_{33}x_{3}^{2}$$

where *y* is the dependent variable, *β* denotes the regression coefficients calculated from the experimental data by multiple regression using the least-squares method, and *x* denotes the independent variables. The experimental data were analyzed by response surface methodology using Statgraphics software (v17.2.00; Statpoint Technologies, Inc., Warrenton, VA, USA).

In this study, experimental modeling of PLA and resin degradation was performed using an incomplete 3^3^ factorial design [57] to evaluate the influence of specimen thickness (*x*_1_), NaCl concentration (*x*_2_), and contact time (*x*_3_) on hardness variation (Shore A units) and moisture content (dependent variables y_1_–y_8_). The ranges of the independent and dependent variables are shown in Table 1.

The standardized dimensionless independent variables, with coded variation limits from −1 to +1, were defined as *x*_1_ (coded specimen thickness), *x*_2_ (coded sodium chloride concentration), and *x*_3_ (coded exposure time). The correspondence between coded and uncoded variables was established using linear equations derived from their respective variation limits, according to Equation (5) [55].

(5) $$x_{i}=\left(\frac{z_{i}-z_{i}^{0}}{{∆z}_{i}}\right)\beta_{d}$$

where *ΔXi* is the distance between the real value at the center point and the real value at the upper or lower level of a variable; $\beta_{d}$ is the major coded limit value in the matrix for each variable; and $z_{i}^{0}$ is the real value at the central point. Coded variables are then assigned values of −1, 0 and +1, corresponding to the lowest, central and maximum variation limits for each variable (Table 1). Thus, the response surface obtained from coded variables is not influenced by the magnitude of each variable and enables all factors to be combined on a dimensionless scale.

Model adequacy was evaluated using several statistical indicators, including the coefficient of determination (R^2^), adjusted R^2^, standard error of estimate, Durbin–Watson statistic, and ANOVA-based F-ratio and *p*-values. The replicated center points were used to estimate experimental error. Only terms with *p*-values below 0.05 were considered statistically significant and retained in the reduced predictive equations. Models with lower adjusted R^2^ values or Durbin–Watson statistics far from 2 were interpreted with caution, as they may indicate lower predictive reliability or residual autocorrelation.

## 3. Results

### 3.1. Model Statistical Testing

In this study, DoE was performed using a BBD to identify the experimental conditions that optimized the three variables assessed in relation to hardness variation and moisture content. Table 2 shows the experimental conditions for the independent variables, expressed as coded values. It also lists the experimental data obtained for the dependent variables y1–y8, corresponding to hardness and moisture content for PLA_B_, PLA_W_, Std-Resin, and Eco-Resin. The experimental sequence was randomized to reduce the influence of systematic errors on the interpretation of the results. Experiments 1–12 were used to calculate the regression coefficients, whereas experiments 13–15 were center-point replicates used to estimate experimental error.

### 3.2. Comparative Analyses for Hardness and Moisture Variation

The statistical analysis of PLA_B_, PLA_W_, Std-Resin, and Eco-Resin provides an overall view of how material properties respond to specimen thickness (A), NaCl concentration (B), and exposure time (C). Table 3 summarizes the R^2^, adjusted R^2^, standard error, and Durbin–Watson values obtained for the fitted models.

PLA_W_ showed the strongest statistical model for hardness, with an R^2^ value of 97.13% and an adjusted R^2^ of 91.98%, indicating that the experimental factors explained most of the variability in the response. Six factors were statistically significant (*p* < 0.05; Table 4), suggesting that PLA_W_ hardness was highly sensitive to the tested process parameters.

Among the factors tested for PLA_W_ hardness, specimen thickness (A = −145.75) and NaCl concentration (B = −64.5) had negative effects, whereas time (C = 60.75) showed a positive effect. Significant synergistic effects were observed for time-related interactions, particularly the BC interaction, suggesting that the combined effect of salinity and exposure time substantially alters material resistance. The quadratic effect of time (CC = −131.75) was the most prominent nonlinear factor, indicating a peak in hardness followed by a decline over the experimental period. In this context, the second-order terms are relevant because they describe curvature in the response surface and make it possible to detect nonlinear trends, local maxima or minima, and material-specific optimum conditions.

In contrast, PLA_B_ showed a lower explanatory capacity, with R^2^ = 88.30% and adjusted R^2^ = 67.23%, and only two significant factors, indicating a weaker dependence of hardness on the studied variables.

A similar trend was observed when comparing the two resins. Std-Resin showed the highest average hardness among the tested materials and a relatively high goodness of fit. Increasing specimen thickness had a negative effect on hardness, whereas exposure time acted as a strengthening factor. A strong positive interaction was identified between thickness and time, indicating that larger specimens retained or increased their hardness more effectively over time. The model was also influenced by negative quadratic terms for thickness and time, defining a clear parabolic response surface for this resin.

**Figure 1:**
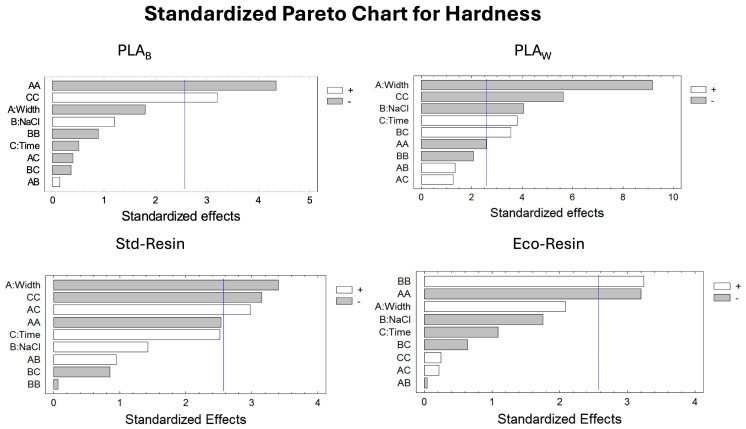
Standardized Pareto charts for the main effects observed on hardness in the PLA and resin materials. The blue vertical line indicates the statistical significance threshold at α < 0.05.

Eco-Resin showed distinct behavior compared with both thermoplastic and standard resin materials. Statistical analysis revealed a moderate predictive capability and two significant factors. According to the standardized Pareto charts (Figure 1), increased thickness had a positive effect on hardness, whereas NaCl concentration and time negatively affected structural integrity. The response was mainly governed by quadratic effects. The quadratic term for NaCl concentration showed a positive effect, whereas the quadratic term for thickness showed a negative effect. Interaction terms were relatively low compared with their standard errors, indicating that the factors acted largely independently in this material. These results suggest that hardness in both resins was moderately affected by the experimental conditions, but less strongly than in PLA_W_.

**Figure 2:**
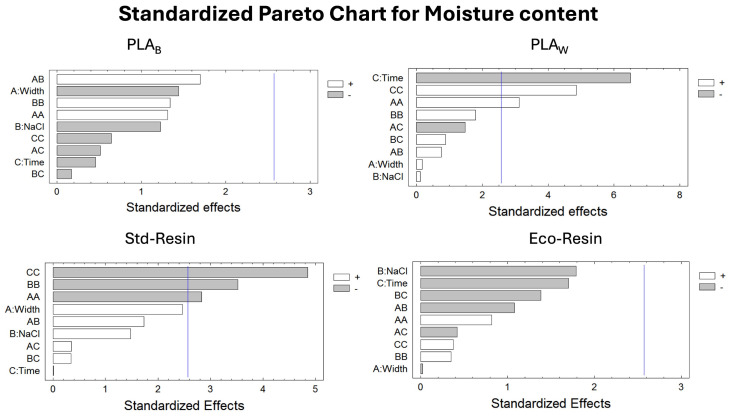
Standardized Pareto charts for the main effects observed on moisture content in the PLA and resin materials. The blue vertical line indicates the statistical significance threshold was set at α < 0.05.

Moisture content showed less predictable behavior. The Pareto charts for PLA_B_ and Eco-Resin suggest that the analyzed factors did not fully explain the variability in moisture uptake (Figure 2). In PLA_W_, the linear effect of time (C) was the dominant factor and clearly exceeded the significance threshold. This was followed by the quadratic effect of time (CC) and the linear effect of thickness (A). Interaction terms such as BC (NaCl–time) and AC (thickness–time) showed moderate influence, whereas the linear contribution of NaCl concentration (B) was relatively small. In Std-Resin, the quadratic term CC showed the largest standardized effect, indicating a strong nonlinear dependence on time. The quadratic terms BB and AA also contributed to the model.

According to the significant factors shown in Table 4, Equations (6)–(11) may be used as predictive models for hardness variation or moisture content in PLA and photopolymer resins as a function of specimen thickness, salinity concentration, and exposure time. The following equations were obtained by excluding coefficients that were not statistically significant at the 95% confidence level (*p* < 0.05).

(6) $$y_{1}=432.33-103.42{\left[width\right]}^{2}+75.96{\left[time\right]}^{2}$$

(7) $$y_{3}=416.0-72.88\left[width\right]-32.25\left[NaCl\right]+30.38\left[time\right]-30.38{\left[width\right]}^{2}-65.88{\left[time\right]}^{2}$$

(8) $$y_{4}=0.002-0.117\left[time\right]+0.083{\left[width\right]}^{2}+0.129{\left[time\right]}^{2}$$

(9) $$y_{5}=276.0-39.0{\left[width\right]}^{2}+39.5{\left[NaCl\right]}^{2}$$

(10) $$y_{7}=470.67-48.88\left[width\right]+60.5\left[width\right]\left[time\right]-66.58{\left[time\right]}^{2}$$

(11) $$y_{8}=0.202-0.056{\left[width\right]}^{2}+0.024{\left[NaCl\right]}^{2}+0.026{\left[time\right]}^{2}$$

### 3.3. Box–Behnken Surface Analyses

**Figure 3:**
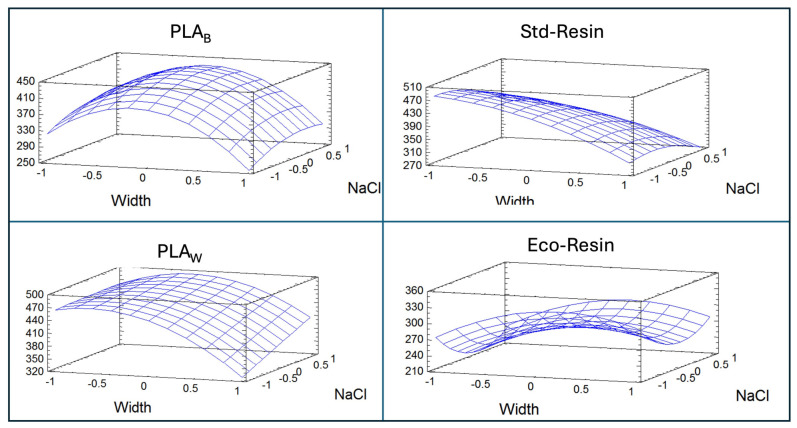
Estimated response surfaces for hardness variation in the four materials tested as a function of NaCl concentration and specimen thickness, with time fixed at an intermediate value.

**Figure 4:**
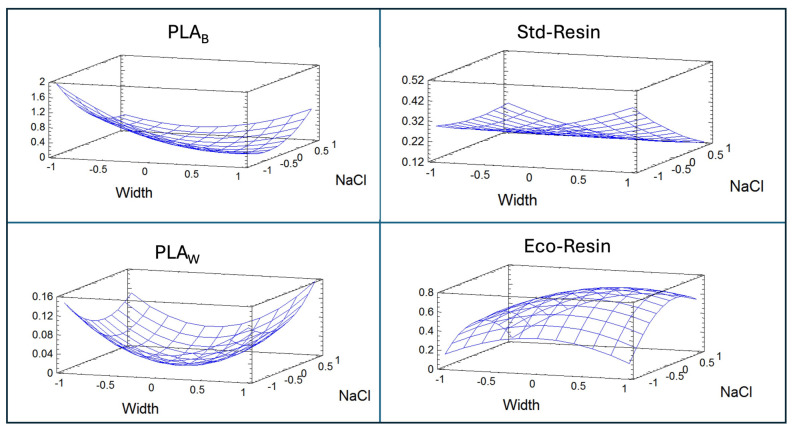
Estimated response surfaces for moisture content variation in the four materials tested as a function of NaCl concentration and specimen thickness, with time fixed at an intermediate value.

Response surface methodology (RSM) enabled visualization of nonlinear interactions among the independent variables. The estimated response surfaces for hardness (Figure 3) and moisture content (Figure 4) were plotted by representing thickness versus NaCl concentration while keeping time at an intermediate value. The response surface plots show that the effects of thickness and NaCl concentration are not purely linear but depend on the material and on the region of the experimental domain. PLA_B_ and Std-Resin showed pronounced curved response surfaces, with the highest hardness values located at intermediate thicknesses and low-to-moderate NaCl concentrations. This curvature indicates that the effect of thickness and salinity on hardness cannot be adequately described by linear terms alone. Instead, the inclusion of quadratic terms in the Box–Behnken models was necessary to capture the presence of local maxima and material-specific optimum regions. Excessive values of both factors tended to reduce hardness, confirming the relevance of second-order effects in describing the surface hardness response. By contrast, PLA_W_ showed a more consistent decrease in hardness as thickness and salt concentration increased, indicating greater sensitivity to environmental degradation. Eco-Resin showed the flattest response surface, suggesting that hardness was less affected by changes in thickness and NaCl concentration than in the other materials.

Regarding moisture absorption, PLA_W_ and PLA_B_ showed U-shaped response surfaces, suggesting that minimum moisture absorption occurred at the central design levels. This behavior further supports the importance of including nonlinear terms, since the response does not follow a simple increasing or decreasing trend across the experimental domain. Std-Resin showed a nearly flat surface with slight increases at the extremes, indicating water absorption stability against moisture uptake. Eco-Resin showed a different pattern, with maximum moisture absorption around the center of the design space and lower values at the extremes of thickness. These results demonstrate that the quadratic model provides a more realistic description of the material responses than a purely linear model, allowing the identification of curvature, local optima, and material-specific degradation trends.

Finally, the fitted models were used to identify the experimental conditions that maximized hardness and minimized water uptake (Table 5). The analysis indicated that exposure time is a key factor affecting hardness. Across the materials, the model predicted optimal hardness at low-to-moderate exposure times (coded range from −1.0 to approximately 0.02), indicating that prolonged exposure may promote deterioration mechanisms.

The effect of NaCl concentration is strongly material-dependent. PLA_B_ and Std-Resin show improved hardness at higher NaCl levels (coded values of 0.651 and 0.999; 4.14% and 5%, respectively). PLA_W_ and Eco-Resin, in contrast, exhibit maximum hardness at the lowest NaCl concentration (coded value −1.0; 0.10%), indicating sensitivity to ionic environments and possible disruption of polymer networks. The width parameter shows a secondary effect, with optimal values located near the central region of the design space. This suggests that while geometric factors influence hardness, their effect is less pronounced than that of chemical and temporal variables.

The optimization of moisture uptake presented a different trend compared to hardness. Within the experimental domain, the model predicts that longer exposure times, with coded values close to +1.0, minimize moisture absorption. However, this result should be interpreted cautiously as a model-derived response under the specific tested conditions, rather than as evidence of a general reduction in water absorption with prolonged exposure. Regarding the influence of NaCl on moisture uptake, PLA_B_ and PLA_W_ display intermediate optimal values, reflecting material-dependent responses to saline exposure. In relation to resins, the model predicts minimum Std-Resin moisture absorption at the highest NaCl level (5%), indicating improved barrier properties under saline conditions, whereas Eco-Resin shows optimal performance at the lowest NaCl concentration (0.10%), confirming its susceptibility to ionic environments. The width factor becomes more significant for moisture absorption than for hardness, particularly in Std-Resin, where larger widths contribute to reduced water uptake under the tested conditions.

## 4. Discussion

This study evaluated the response of various 3D-printed polymers under simplified saline and humid conditions. Among the variables analyzed, exposure time was a relevant factor affecting material behavior. Regarding hardness, the dominant negative quadratic effect of exposure time (CC) indicates a nonlinear response, with hardness tending to decrease after the longest exposure tested in the experimental domain. This behavior is consistent with degradation processes reported for PLA-based materials [58] exposed to humid or saline environments, where extended exposure can induce chain scission and reduce surface mechanical strength [59]. This result suggests that extended exposure may promote structural rearrangements within the material, reducing free volume and limiting water diffusion.

The observed effect of NaCl concentration highlights the material-dependent nature of the response. For PLA_B_ and Std-Resin, higher NaCl concentrations were associated with increased predicted hardness within the tested range. However, this trend should be interpreted as an empirical response of the fitted model rather than evidence of a specific hardening mechanism, since no complementary physicochemical or microstructural analyses were performed. In contrast, PLA_W_ and Eco-Resin showed lower hardness at higher salinity levels, suggesting greater sensitivity to saline exposure under the tested conditions.

Differences in PLA-colored filaments have also been observed by other authors. Arthanari et al. [60] revealed differences observed in white, blue and gray PLA polymers related to their surface roughness. In this study, differences observed between blue and white PLA can be partially explained by the influence of pigments and additives on the physicochemical properties of the material. Previous research has shown that PLA filaments of different colors exhibit significant variations in rheological behavior, chemical composition, and thermodynamic properties. Blue and white PLA have been reported to present relatively low flowability, with substantial differences in viscosity at zero shear rate depending on pigment composition [61]. In addition, Wittbrodt and Pearce [62] found that white material exhibits higher crystalline regions (5.05%) among most of the colors and higher than blue filament (4.85%), which supports the idea that color is not just an esthetic parameter in PLA materials, but a factor that significantly influences rheological, structural, and surface mechanical properties, thereby affecting both experimental outcomes and the quality of statistical modeling.

In regard to the resins, particularly the standard resin, a good model fit is observed for hardness, indicating a more predictable response to the experimental factors. However, the Eco-Resin presents a very low Durbin–Watson value, indicating strong autocorrelation in the residuals. This suggests that the model does not fully satisfy the statistical assumptions, possibly due to formulation heterogeneity or unaccounted systematic effects. In practical terms, low moisture uptake is desirable because it reduces the risk of swelling, plasticization, dimensional changes, and loss of comfort during extended contact with sweat or cleaning solutions. Similarly, the retention of Shore A hardness indicates that the material preserves its resistance to indentation and its ability to provide mechanical support. Materials showing both low moisture absorption and stable hardness values can be considered more suitable for long-term orthotic applications. In this sense, Std-Resin exhibited the most favorable durability profile, as it maintained relatively high hardness and limited moisture uptake under the tested saline conditions. By contrast, PLA-based materials and Eco-Resin showed greater sensitivity to saline exposure and immersion time, suggesting that their use in orthotic devices may require optimized thickness, protective coatings, or controlled cleaning protocols.

The influence of resin nature on mechanical properties has been little studied so far. Miller-Schulze and Williams [63] found differences between eco and standard photopolymer resins in terms of emissions of volatile organic compounds, particularly reactive monomers such as 2-hydroxyethyl acrylate and 4-acrylomorpholine, compared to eco formulations. These differences in chemical profiles may be related to formulation-dependent behavior, although monomer composition, crosslinking density, and network structure were not directly analyzed in the present study. Instead, Stefaniak et al. [64] found that color exhibits little influence in the resin behavior, but printer brand, printer configuration and resin brand are other factors that can cause significant variations in the behavior of different materials.

On the other hand, the relatively minor role of specimen width in hardness optimization, compared to chemical and temporal variables, suggests that geometric factors primarily influence structural integrity indirectly, for example through porosity or internal stress distribution. However, its more pronounced effect on moisture absorption, particularly in Std-Resin, indicates that increased width may reduce permeability by limiting diffusion pathways. This highlights that selection and prediction of processing parameters can have great relevance [65], reinforcing the need of optimization approaches.

From a materials perspective, the comparative analysis indicates that Std-Resin offers the most balanced properties, maintaining relatively high hardness while exhibiting low moisture absorption under specific conditions. PLA_W_ demonstrated higher sensitivity to environmental factors, which may limit their applicability in aggressive conditions unless further modified. Eco-Resin, while environmentally attractive, shows the greatest susceptibility to both salinity and time, suggesting that additional formulation improvements may be necessary to enhance its stability.

Despite the relevance of the results, this study has some limitations. The experiments were conducted under controlled in vitro saline conditions and therefore do not fully reproduce real orthosis use, where variable sweat composition, pH, temperature, mechanical loading, cleaning procedures, friction, and wet–dry cycles may occur. Moreover, only moisture absorption and Shore A hardness were evaluated, while fatigue resistance, dimensional stability, surface degradation, aging behavior, and biocompatibility were not assessed. Thus, the findings should be interpreted as an initial comparative assessment under defined saline conditions. Future studies should include more realistic sweat formulations, cyclic loading, longer aging periods, and user-simulated or in vivo validation.

The results highlight the importance of combining material selection with predictive modeling when designing 3D-printed orthotic components intended for extended skin contact. The differences observed among PLA, Std-Resin, and Eco-Resin indicate that material chemistry and processing route influence both hardness retention and moisture uptake under saline exposure. Std-Resin showed the most stable surface hardness response, which supports its use when dimensional and mechanical stability are the main design requirements. However, PLA and Eco-Resin remain attractive from a sustainability perspective if thickness, exposure conditions, and maintenance protocols are carefully optimized. The BBD approach was useful for identifying nonlinear effects and material-specific responses, but the interpretation of optimum conditions should be considered together with clinical requirements, comfort, hygiene, and extended use under controlled saline exposure conditions. Additional validation with cyclic loading, artificial sweat formulations, and longer exposure periods would strengthen the translation of these findings to real orthotic applications.

## 5. Conclusions

This study evaluated the resilience of several 3D-printed polymers under saline and humid conditions to approximate controlled saline exposure conditions related to sweat and hygiene-related contact. The results indicate that Standard Blue Resin was the most suitable option for applications requiring high-dimensional and surface hardness stability, whereas PLA and Eco-Resin showed greater sensitivity to moisture uptake and saline exposure. Polymeric sheet thickness was a relevant design factor, particularly in thinner specimens, where plasticization and softening may compromise mechanical performance. NaCl concentration also affected material behavior in a material-dependent manner, confirming the need to define cleaning protocols and, where necessary, protective coatings for long-term wearable orthoses. The Box–Behnken response surface models provided a useful predictive framework for identifying material-specific optimum conditions and for supporting the design of biomimetic orthotic devices that balance durability, comfort, and sustainability.

## Figures and Tables

**Table 1:** Independent and dependent variables used in the Box–Behnken experimental design.

(a) Independent variables			
Variable	Nomenclature	Units	Variation range
Specimen width	[Width]	mm	2–6
Sodium chloride percentage	[NaCl]	%	0.1–5
Time	[t]	days	1–6
(b) Dimensionless, coded independent variables			
Variable	Nomenclature	Definition	Variation range
Dimensionless specimen width	x_1_	([Width] − 4)/2	(−1, 1)
Dimensionless sodium chloride percentage	x_2_	([NaCl] − 2.5)/2.4	(−1, 1)
Dimensionless time	x_3_	([t] − 3.5)/2.5	(−1, 1)
(c) Dependent variables			
Variable	Nomenclature	Units	
Blue PLA hardness	y_1_	Shore A units	
Blue PLA moisture content	y_2_	%	
White PLA hardness	y_3_	Shore A units	
White PLA moisture content	y_4_	%	
Eco-Resin hardness	y_5_	Shore A units	
Eco-Resin moisture content	y_6_	%	
Standard resin hardness	y_7_	Shore A units	
Standard resin moisture content	y_8_	%	

**Table 2:** Operational conditions considered in this study, expressed as coded independent variables, and experimental results obtained for the dependent variables analyzed.

Independent Variables				Dependent Variables							
				Blue PLA		White PLA		Eco-Resin		Standard Resin	
Exp.	x_1_	x_2_	x_3_	y_1_	y_2_	y_3_	y_4_	y_5_	y_6_	y_7_	y_8_
1	0	−1	−1	462	0.303	362	0.301	319	0.101	321	0.321
2	0	1	−1	531	0.5	206	0.302	311	0.102	384	0.432
3	0	−1	1	460	0.224	366	0.01	341	0.002	456	0.251
4	0	1	1	495	0.222	370	0.101	303	0.101	450	0.001
5	−1	−1	0	298	2.6	488	0.201	263	0.003	430	0.421
6	−1	1	0	317	0.501	405	0.125	229	0.004	445	0.301
7	1	−1	0	292	0.321	288	0.101	325	0.203	348	0.402
8	1	1	0	325	0.201	265	0.102	289	0.701	440	0.001
9	−1	0	−1	462	0.203	376	0.25	260	0.103	465	0.21
10	−1	0	1	466	0.303	385	0.103	212	0.101	388	0.201
11	1	0	−1	363	0.605	226	0.4	263	0.105	192	0.42
12	1	0	1	330	0.105	292	0.102	225	0.204	357	0.301
13	0	0	0	476	0.101	418	0.001	279	0.701	454	0.201
14	0	0	0	417	0.102	416	0.002	275	0.702	484	0.202
15	0	0	0	404	0.103	414	0.003	274	0.705	474	0.203

**Table 3:** Summary of the quadratic models obtained from the Box–Behnken experimental design for the studied materials and response variables. The table includes the coefficient of determination, adjusted coefficient of determination, standard error, and Durbin–Watson statistic.

Material	Variable	R^2^ (%)	Adjusted-R^2^ (%)	Standard Error	Durbin–Watson
Blue PLA	Hardness	88.29	67.23	45.42	1.90
	Moisture	68.64	12.20	0.58	1.56
White PLA	Hardness	97.13	91.98	22.49	2.53
	Moisture	94.01	83.22	0.051	1.34
Eco-Resin	Hardness	86.34	61.75	23.41	0.12
	Moisture	86.34	61.75	23.41	1.66
Standard resin	Hardness	88.29	67.23	45.42	1.90
	Moisture	68.64	12.20	0.58	1.56

**Table 4:** Analysis of variance (ANOVA) results for the fitted quadratic models describing hardness and moisture content of blue PLA, white PLA, standard resin, and Eco-Resin (A = thickness; B = NaCl concentration; C = time). Only statistically significant effects (*p* < 0.05) are shown.

		Source	Sum of Squares	df	Mean Square	F-Ratio	*p*-Value
Hardness	Blue PLA	AA	39,203.40	1	39,204.40	19.01	0.0073
		CC	21,303.40	1	21,303.40	10.33	0.0236
	White PLA	A	42,486.1	1	42,486.1	83.97	0.0003
		B	8320.5	1	8320.5	16.45	0.0098
		C	7381.13	1	7381.13	14.59	0.0124
		AA	3406.67	1	3406.67	6.73	0.0486
		BC	6400.0	1	6400.0	12.65	0.0163
		CC	16,022.8	1	16,022.8	31.67	0.0025
	Standard resin	A	19,110.1	1	19,110.1	11.57	0.0192
		AC	14,641.0	1	14,641.0	8.86	0.0309
		CC	16,369.3	1	16,369.1	9.91	0.0254
	Eco-Resin	AA	5616.0	1	5616.0	10.25	0.0240
		BB	5760.92	1	5760.92	10.51	0.0229
Moisture	Blue PLA	--	--	--	--	--	--
	White PLA	C	0.109746	1	0.109746	42.17	0.0013
		AA	0.02528	1	0.02528	9.72	0.0263
		CC	0.0614	1	0.0614	23.61	0.0046
	Standard resin	--	--	--	--	--	--
	Eco-Resin	--	--	--	--	--	--

**Table 5:** Predicted optimal conditions for maximizing hardness and minimizing moisture absorption in the materials analyzed.

	Maximize Hardness				Minimize Moisture Absorption			
Coded	PLA_B_	PLA_W_	Std-Resin	Eco-Resin	PLA_B_	PLA_W_	Std-Resin	Eco-Resin
Width	−0.0853	−1.0	−0.264	0.199	0.531	0.109	0.891	−1.0
NaCl	0.651	−0.999	0.999	−1.0	0.0492	−0.159	1.0	−1.0
Time	−1.0	−0.181	0.0221	−0.999	1.0	0.484	0.999	0.999
Uncoded	PLA_B_	PLA_W_	Std-Resin	Eco-Resin	PLA_B_	PLA_W_	Std-Resin	Eco-Resin
Width (mm)	3.83	2.00	3.47	4.40	5.06	4.22	5.78	2.00
NaCl (%)	4.14	0.10	5.00	0.10	2.67	2.16	5.00	0.10
Time (days)	1.00	3.05	3.56	1.00	6.00	4.71	6.00	6.00

## Data Availability

The datasets generated and/or analyzed during the current study are publicly available in the Zenodo repository at https://zenodo.org/records/19917788 (accessed on 30 April 2026).
